# The distribution and behavioral characteristics of plateau pikas (*Ochotonacurzoniae*)

**DOI:** 10.3897/zookeys.1059.63581

**Published:** 2021-09-14

**Authors:** Jun Qiu, Cang Ma, Ying-Hui Jia, Jin-Zhao Wang, Shou-Kai Cao, Fang-Fang Li

**Affiliations:** 1 State Key Laboratory of Plateau Ecology and Agriculture, Qinghai University, Xining, 810016, China Qinghai University Xining China; 2 State Key Laboratory of Hydroscience & Engineering, Tsinghua University, Beijing, 100084, China Tsinghua University Beijing China; 3 School of Water Resources and Electric Power, Qinghai University, Xining, 810016, China China Agricultural University Beijing China; 4 College of Water Resources & Civil Engineering, China Agricultural University, Beijing, 100083, China Qinghai Universit Xining China

**Keywords:** Dari County, field observations, population density, Qinghai-Tibet Plateau (QTP), random encounter model (REM)

## Abstract

Plateau pikas (*Ochotonacurzoniae*) are regarded as one of the main causes of the degradation of alpine meadows in the Qinghai-Tibet Plateau (QTP). The population density of plateau pikas is directly related to the degree of grassland damage. In this study, field observation was conducted for one week in the southeastern QTP in August 2019. A random encounter model (REM) was used to estimate the population density of plateau pikas from photographs and videos, and the frequencies of different behaviors were calculated. In addition, the effects of water-source distance and terrain on the distribution of plateau pikas and the frequencies of different pika behaviors under different population densities were explored. The observations and knowledge derived from this study provide a reference for the population control of plateau pikas.

## Introduction

The Qinghai-Tibet Plateau (QTP) is the highest plateau in the world, with an average altitude of 4500 m, and it is known as the roof of the world. Alpine meadows are widespread in the QTP, accounting for more than 50% of the total area of the QTP (Gao et al. 2013; Chen et al. 2014). Such meadows are especially widespread in the source areas of the Yangtze, Yellow, and Lancang Rivers. Alpine meadows play very important roles in forage production, water conservation, climate regulation, biodiversity maintenance and so on (Chen et al. 2008; Wen et al. 2013; Yao et al. 2019). Due to the special climatic conditions and natural environment of the QTP, the QTP ecosystem is fragile. Once the habitat is destroyed, it is difficult to recover, and recovery takes a long time (Schleuss et al. 2015; Guo et al. 2019). To date, the degraded area of alpine meadows in the QTP has reached 4.67 × 10^6^ hm^2^, accounting for 25% of the total area of the region; moreover, 50% of the total alpine meadow area, 2.13 × 10^6^ hm^2^, comprises black-soil-type degraded grasslands (Dong et al. 2013). This “black soil beach” grassland is the product of severe degradation of alpine meadow grassland and has extremely low production capacity. The appearance of bald patches is a prominent feature of these black soil beaches and drives their formation (Gao and Li 2016).

In recent years, the black soil beach area has continued to expand, and there are few countermeasures for this problem. Degraded alpine meadows seriously affect the sustainable development of the ecological environment and animal husbandry in the QTP (Gao et al. 2019). Previous studies have indicated that overgrazing, rampant rodent damage, climate change and glacier retreat are the main reasons for the degradation of alpine meadows in the QTP (Chen et al. 2017). Rodent damage is mainly caused by the plateau pika, *Ochotonacurzoniae* (Hodgson, 1858). This small non-hibernating herbivore belongs to the family Ochotonidae. In the QTP, plateau pikas are mainly distributed in areas with altitudes of 3200~5300 m and live in groups (Smith et al. 2010; Koju et al. 2017). A family of plateau pikas consists of 2~6 adult pikas and their offspring (Pan and Migmar 2016; Wei et al. 2019). Due to their strong survival and reproductive abilities, they are widely distributed in the QTP, and their population density can reach more than 300 individuals/ha. Plateau pikas burrow in and dig soil, gnaw at grass and roots, and destroy the grass layer. In addition, long-term overgrazing creates favorable conditions for plateau pika invasion, thereby accelerating grassland degradation (Bai et al. 2002). According to Fan et al.’s study (Fan 1999), the annual consumption of forage by plateau pikas is approximately 150 × 10^6^ t, equivalent to that of 150 × 10^6^ grazing sheep a year. Therefore, it is important to control the number of pikas to reduce grassland degradation.

Controlling population density is the key to animal management and protection (Qu et al. 2017). The population density of rodents is directly related to the degree of grassland damage; higher numbers of rodents cause more serious damage. Plateau pikas are a key species on the QTP and play an irreplaceable role in maintaining the balance and stability of grassland ecosystems (Smith et al. 2019). Controlling the population of plateau pikas is a fundamental way to implement grassland protection, which is facilitated by understanding the distribution and behavior patterns of plateau pikas. Many studies on this topic have been completed. Qu et al. (2017) found that the population of plateau pikas exhibits almost no interyear changes but exhibits intrayear variation, with population numbers peaking in June. They also demonstrated that survival of plateau pikas experience seasonal fluctuations, while demographic features and climate together regulate population dynamics. Since the altitude of the QTP ranges from 3000 to 5000 m, the population dynamics and population peaking of plateau pikas may differ between regions with different altitudes and climates.

Some researchers are also concerned with the behavioral characteristics of plateau pikas. Smith et al. (1986) has categorized different pika activities into social behaviors (such as chasing, playing and following) and nonsocial behaviors (such as eating, vigilance and digging). Wang et al. (2005) investigated the varied behavioral patterns with different population densities, and discovered that the behavior change of plateau pika was obviously related to its breeding period and sex. It is also reported that plateau pikas reduce the risk of predation by adjusting behavioral strategies (Wei et al. 2004). Therefore, the behavioral patterns of plateau pikas change with different population densities and external environments to enable better survival.

Wei et al. (2003) indicated that the population size of plateau pikas differed significantly among grasslands with varying degrees of degradation and increased with the aggravation of grassland degradation; in addition, they found that the population size was highest in moderately degraded grasslands and decreased in severely degraded grasslands due to a lack of food. Wangdwei (2019) used Poisson regression to analyze the effects of yaks and land use type on plateau pika behavior. The results indicated that the frequency of foraging behavior was higher than that of warning behavior in winter but lower than that of warning behavior in summer, and that the level of vegetation coverage was inversely proportional to pika foraging frequency. Such knowledge helps us better understand the relationship between plateau pikas and grassland degradation.

In this study, field observation was conducted for one week in the southeastern QTP from August 12 to 18, 2019. The random encounter model (REM) established by Rowcliffe et al. (2008) was used to estimate the population density of plateau pikas from photographs and videos, and the frequencies of different behaviors were calculated. In addition, the effects of water-source distance and terrain on the distribution of plateau pikas and the frequencies of different pika behaviors under different population densities were explored. This study contributes to the ecological knowledge on plateau pikas and provides a reference for the control of plateau pikas.

## Methods

### Study area

The source area of the Yellow River is located in the southern portion of the Qinghai Province and has a total area of 137.7 × 10^3^ km^2^, of which grassland accounts for 81.2%. Alpine grassland and alpine meadows are typical vegetation types in the source region of the Yellow River (Li et al. 2016). The source area of the Yellow River has been the habitat of many wild animals and the grazing area of livestock for a long time. However, in recent decades, due to environmental changes driven by rodent and human activities and other adverse factors, the ecological environment of the Yellow River source area has deteriorated, resulting in severe grassland degradation.

Dari County is located in the southern portion of the Yellow River source area (32°36'42"~34°15'20"N, 98°15'29"~100°32'41"E). The county has an average altitude of 4426 m and an alpine semihumid climate. There is no obvious division of the year into four seasons; rather, there are cold and warm seasons. There are cold monsoons and heavy snow in winter, which lasts for up to 7–8 months. The warm season is humid but lasts for only 4–5 months. The average temperature of the county is between -0.1 °C and -3.5 °C. The annual precipitation is approximately 560 mm and mostly occurs from June to September. There are only a few grassland types, with most grasslands being alpine meadow grasslands. The grasslands begin to turn green in mid-May, and the annual growth period is only 120 days.

There are 1,402 million hectares of natural grasslands in Dari County, accounting for 94% of the county’s total land area, and 1,117 million hectares of usable grasslands, accounting for 80% of the total natural grassland area. In recent decades, the grasslands in Dari County have undergone continuous degradation, with the area of moderately degraded grasslands reaching 50%–60% of the total usable grassland area (Zhang et al. 2011). The black soil beach area, which has extremely low utilization value, is expanding, rodent infestation is rampant, and vegetation patches are widespread; therefore, Dari County has become the county with the most severe grassland degradation in the source region of the Yellow River (Liu et al. 2008). Due to its significance, Dari County was selected as the study area.

### Field observation

**Figure 1.**
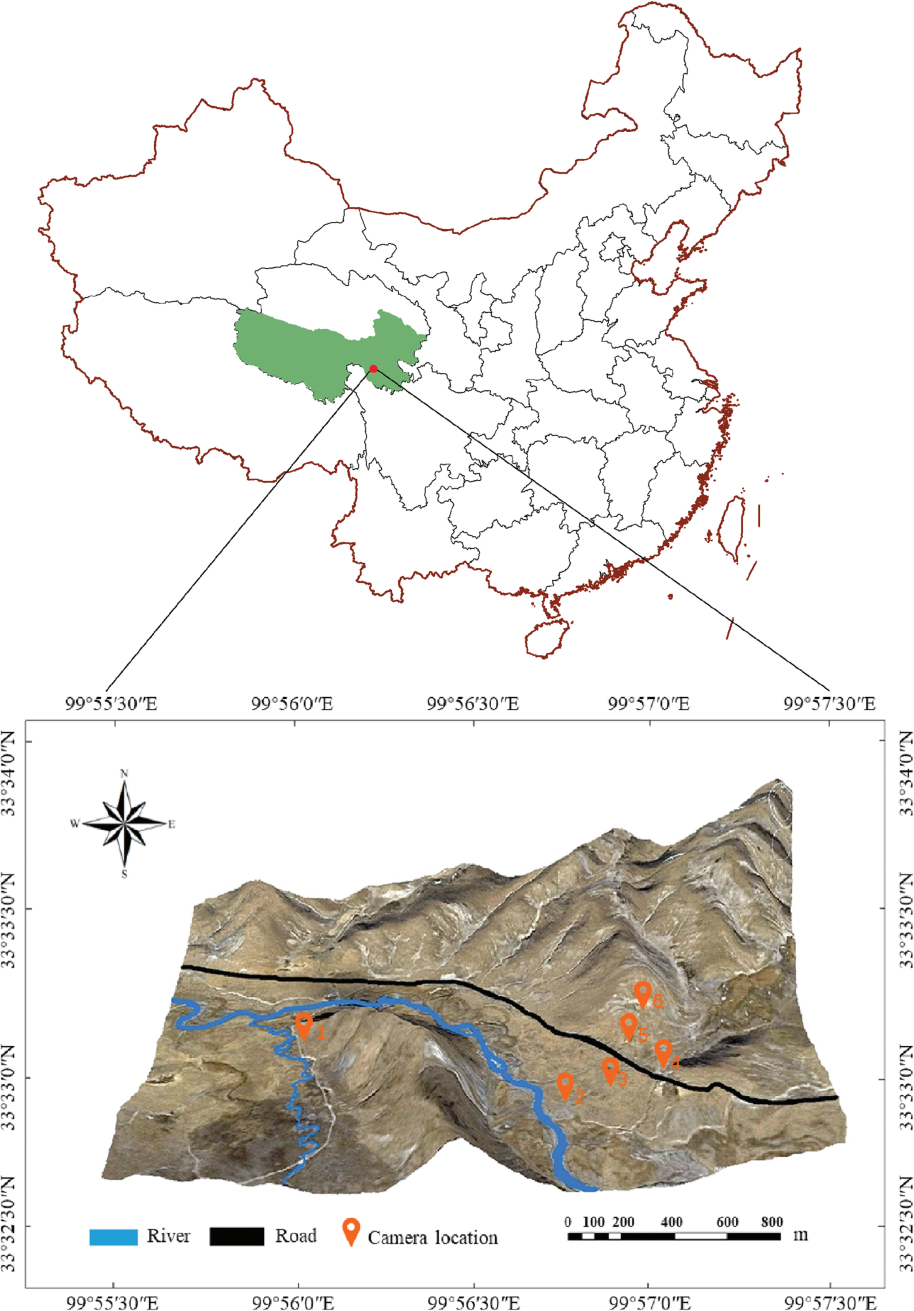
Infrared camera locations.

Camera trap technology has been widely used for wildlife population density estimation and behavior observation due to its low labor cost, minimal interference with the environment and strong adaptability. The camera used in this study was the Foresafe H885 field infrared camera, as shown in Fig. 1. In the layout, the camera was oriented so that the lens was approximately parallel to the ground to avoid direct sunlight on the lens. The front view of the camera was cleared, without occlusion, and the scene environment restored once the camera was installed. Deployment time and other local information, such as GPS coordinates, were recorded. Six cameras numbered #1 to #6 were arranged at six different locations differing in water-source distance and terrain. Camera #1 was installed in the floodplain; cameras #2, #3 and #4 were placed 100 m, 300 m and 600 m, respectively, from the river channel; and cameras #5 and #6 were placed on a steep slope and gentle slope, respectively. The camera locations are shown in Fig. 1.

The six cameras were installed from August 12 to August 18, 2019. The infrared sensor on each camera could actively detect sudden changes in infrared energy in the field of view, triggering the activation of the camera. Each camera was operational 24 h a day in photo and video modes, and the video recording time was set to 30 s. Once triggered, the camera took a photograph, recorded 30 s of video, and then returned to standby mode. The time, temperature and other information associated with the images and videos were stored on the memory card in chronological order.

With the aim of reducing the autocorrelation of the field observation photographs, two photographs were considered independent if the time interval between them was more than two minutes. The camera trigger time, temperature, frequencies of plateau pika behaviors and other data from the videos were recorded.

### Estimation of population density

**Figure 2.**
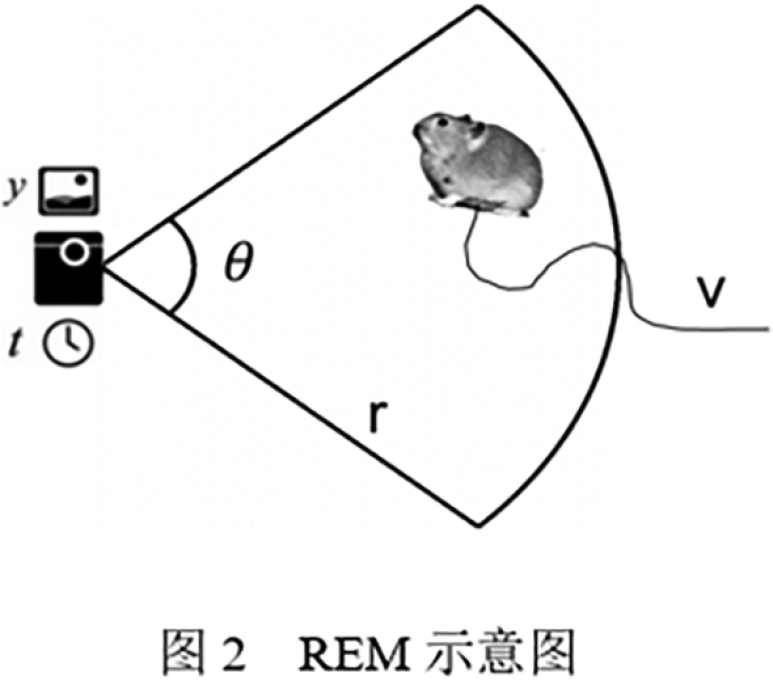
Schematic diagram of the REM parameters.

The REM proposed by Rowcliffe et al. (2008) was adopted in this study to estimate population density from the camera trap data; this approach does not require individual recognition of animals. Since it was proposed, the REM has been applied to estimate the population density of many animal species (Manzo et al. 2012; Anile et al. 2014; Caravaggi et al. 2016) and is shown in the following equation:

$D=\frac{y}{t}\frac{\pi }{vr(2+\theta )}$,

where *D* is population density; *y* is the number of independent images; *t* is the number of days the camera was operational; *v* is the daily movement speed of animals; *r* is the radius of the camera detection area; and *θ* is the camera detection angle, as shown in Fig. 2.

Preliminary experiments revealed that the detection information of each camera was r = 8 m and *θ* = 55° (0.96 rad). According to Zhang et al. (Zhang et al. 2013), the daily travel distance of plateau pikas is approximately 300 m; thus, *v* was set to 0.3 km/d. The total number of independent images (*y*) was obtained by counting the number of images taken by each camera.

### Classification of pika behavior

**Figure 3.**
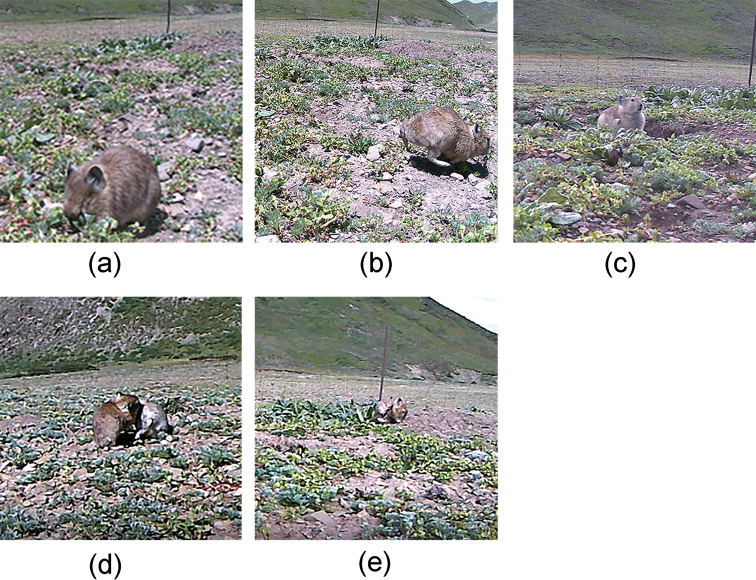
Classification of plateau pika behavior **a** foraging **b** traveling **c** being vigilant **d** fighting; and **e** grooming.

According to the literature and obtained video data, the behavior of plateau pikas was classified into five types: foraging, traveling, being vigilant, grooming and fighting, as described and illustrated in Table 1 and Fig. 3.

**Table 1.** Classification of plateau pika behavior.

Behavior	Description
Foraging	Consuming food while in place or collecting food while moving
Traveling	Moving quickly from one place to another
Being vigilant	Siting on the ground with neck extended or standing with the forefeet off the ground
Fighting	Aggressively grabbing and biting another pika
Grooming	Cleaning the body with the paws or mouth

## Results

### Population density

During the one week of field observation, a few of the cameras did not function properly on some days due to displacement and other reasons. A total of 1138 independent images were obtained. The working days of the camera at each position, the total number of independent images taken, and the population density of plateau pikas estimated by the REM equation are presented in Table 2. The results showed that the average density of plateau pikas in the study area was 144 per hectare, the highest density was 200 individuals/ha, at 100 m along the riverbank, and the lowest density was 77 individuals/ha, on the sunny side of the steep slope.

**Table 2.** Population density of plateau pikas estimated by REM at different locations.

Location	Number of camera working days	Number of independent images	Number of plateau pikas per *ha*
Riverbank	5	161	142
100 m from riverbank	6	271	200
300 m from riverbank	7	270	171
600 m from riverbank	4	130	144
Sunny side of gentle slope	7	201	127
Sunny side of steep slope	6	105	77
**MEAN population density**			**144**

The distance from the water source had a strong impact on the density of plateau pikas, which was highest at the location 100 m from the riverbank, followed by the locations 300 m from the riverbank, 600 m from the riverbank, and on the riverbank. In addition, the density of plateau pikas was significantly higher on the sunny side of the gentle slope than on that of the steep slope.

### Frequencies of different behaviors at different locations

**Figure 4.**
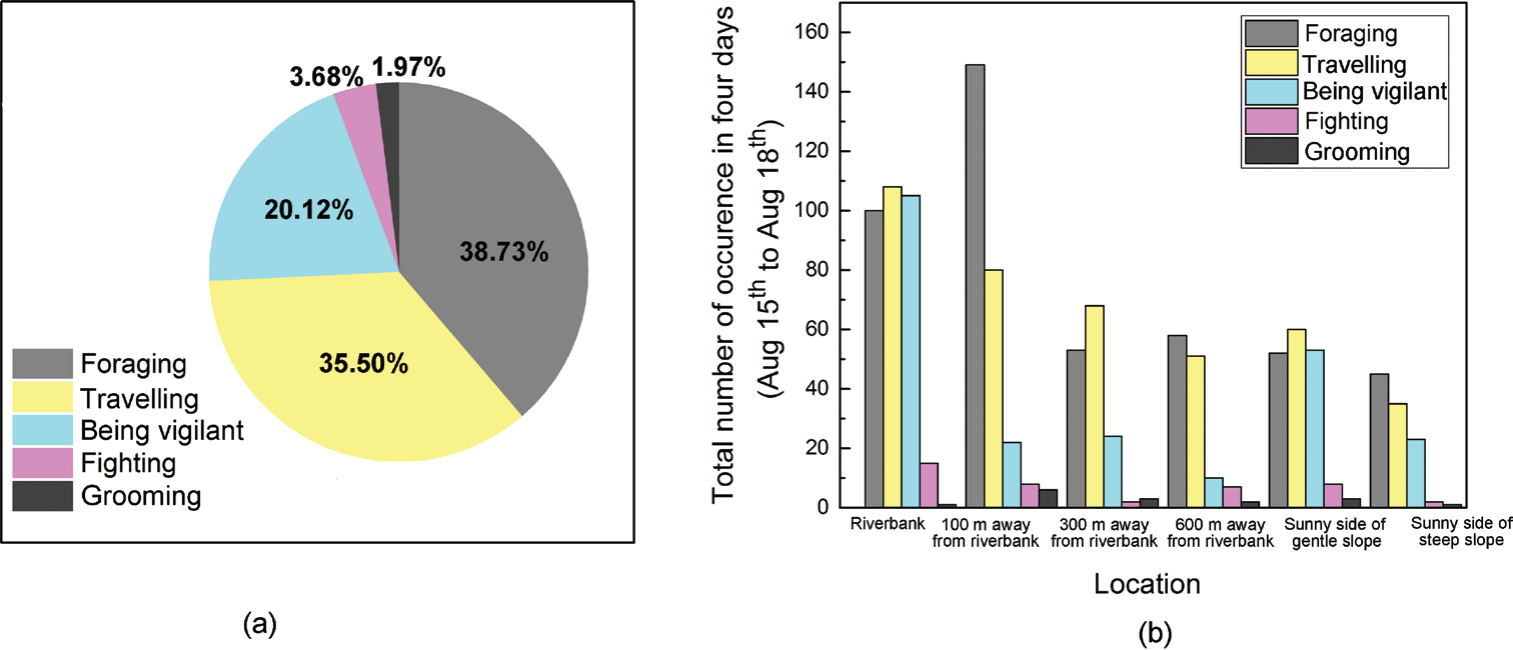
Frequencies of the five behaviors defined in Table 1 and Fig. 3**a** percentages of occurrence of the different behaviors and **b** total number of occurrences of different behaviors in different locations.

From August 15 to August 18, all six cameras functioned normally. The frequencies of the five behaviors defined in Table 1 and Fig. 3 in the different locations are shown in Fig. 4.

As shown in Fig. 4a, foraging and traveling behaviors accounted for the largest proportions of plateau pika behavior, with percentages of 38.73% and 35.50%, respectively; vigilance behavior accounted for 20.12% of the behavior, and fighting and grooming accounted for only 3.68% and 1.97%, respectively. Figure 4b indicates that the frequency of foraging behavior was highest 100 m from the riverbank and that the frequencies of vigilance behavior and traveling were highest on the riverbank. Compared with that at the riverbank, the frequency of vigilance behavior was significantly lower at the other locations, and the frequencies of all behaviors were lower on the sunny side of the steep slope than on the sunny side of the gentle slope. Foraging and traveling make up a large part of the pika’s ground activities, which is consistent with findings of Smith et al. (1986). Thus, it can be inferred that the main purpose for pikas to go out is to seek food, with constant moving to find a better and safer place to eat. To reduce the risk of predation, pikas may increase their running and vigilance frequencies, resulting a relatively large proportion in the two behavioral patterns. It is shown in the camera records that pikas living 100 m away from riverbank exhibit the greatest chance of foraging, which may result from better food supplies and larger population density.

### Frequencies of different behaviors during different periods

**Figure 5.**
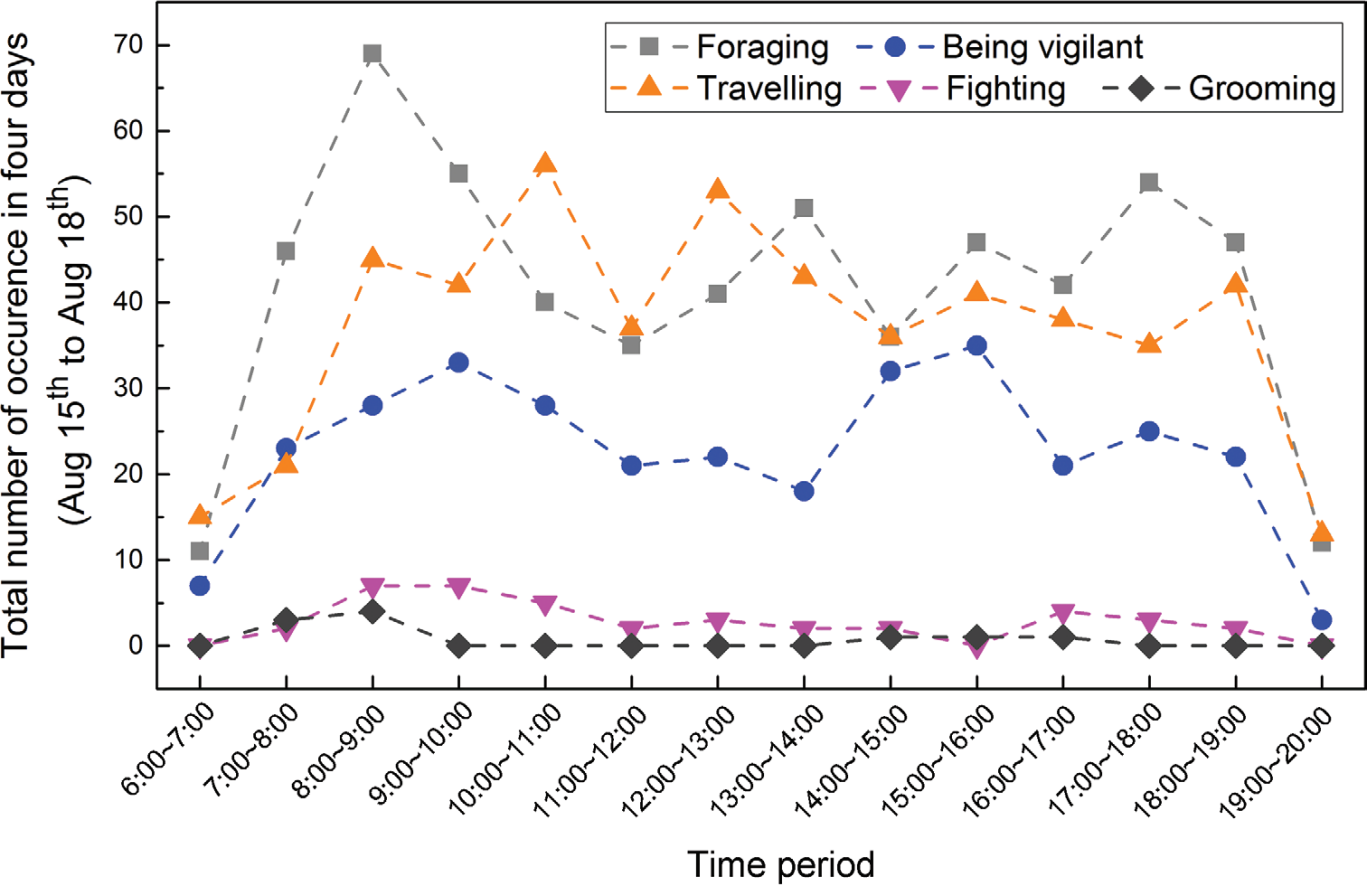
Total number of occurrences of different behaviors during different time periods.

To investigate behavior differences among different time periods, the frequencies of the different behaviors during the time periods of 6:00–7:00, 7:00–8:00,…., and 19:00–20:00 were calculated and are shown in Fig. 5. Two peaks of foraging behavior occurred, at 8:00–9:00 and 17:00–18:00. From 6:00 to 9:00, foraging, vigilance and traveling behaviors gradually increased in frequency, and after 18:00, foraging and vigilance behaviors decreased significantly. The Pearson correlation analysis revealed a significant positive correlation between foraging and vigilance behavior (r = 0.734, P = 0.003).

**Figure 6.**
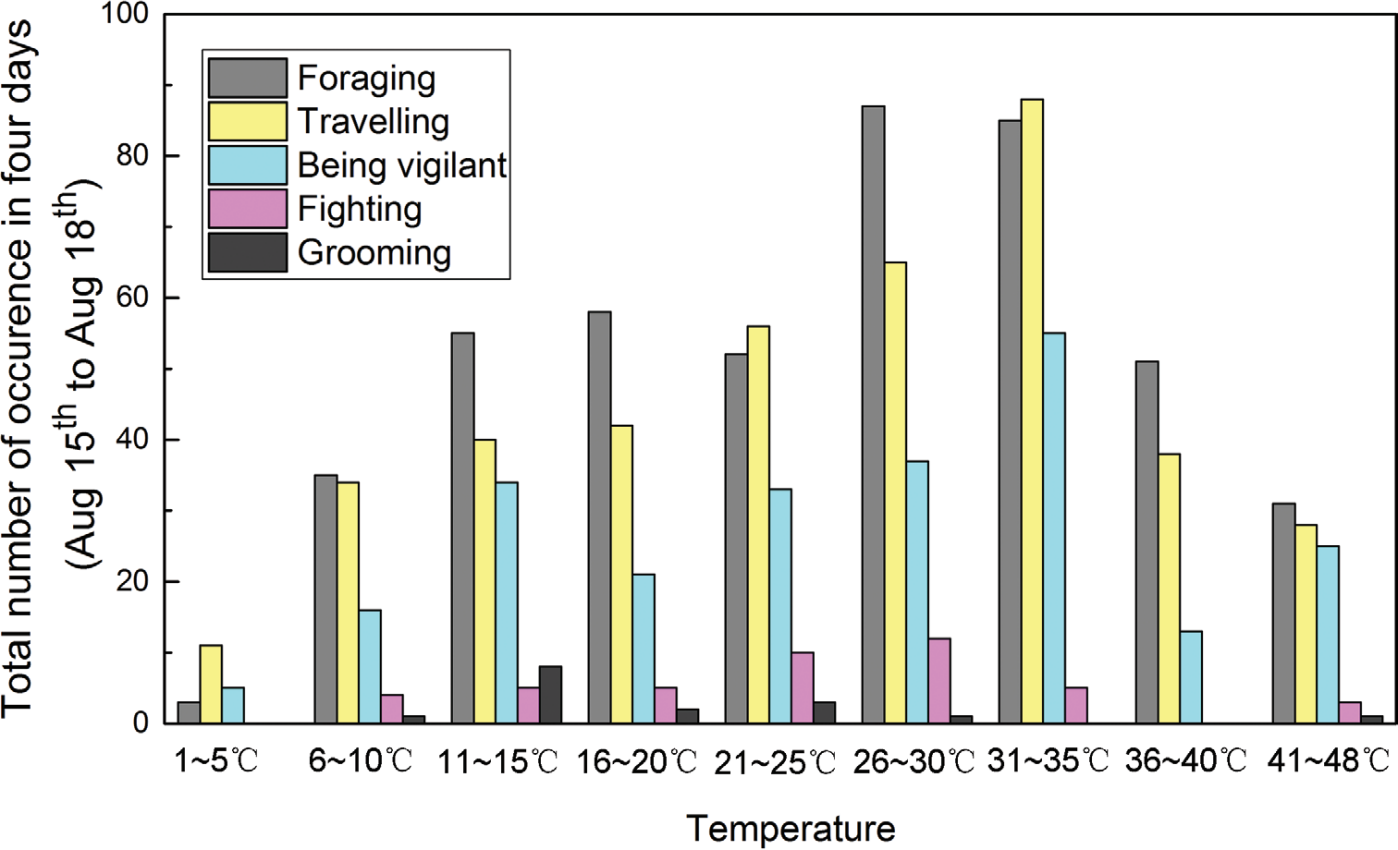
Total number of occurrences of different behaviors under different temperature gradients.

The highest surface temperature recorded by the infrared cameras was 48 °C, and the surface temperature was above 0 °C throughout the observation period. The frequency of each behavior under different temperature gradients is shown in Fig. 6. With increasing temperature, the frequencies of foraging, vigilance and traveling behaviors of plateau pikas increased gradually until 35 °C, decreasing significantly thereafter.

Observations have shown that the period when plateau pikas are active is mainly influenced by light intensity (Hao et al. 1987). It is reported that pikas start their ground activities after daybreak and almost disappear on the ground at night. This may explain the results of Fig. 5, and the preferable temperature for pikas may be around 31~35 °C according to Fig. 6.

### Correlation between density of plateau pikas and area of barren patches

According to Ma’s study (Ma 2006), plateau pikas tend to occupy open habitats with low vegetation coverage. Moderately degraded grassland with sparse vegetation is conducive to pika survival. Studies have shown that among grassland types, moderately degraded grasslands have the highest population densities of pikas. As the pika population increases, the vegetation in the habitat gradually decreases, and the proportion of bare land area increases, which leads to more severe grassland degradation.

**Figure 7.**
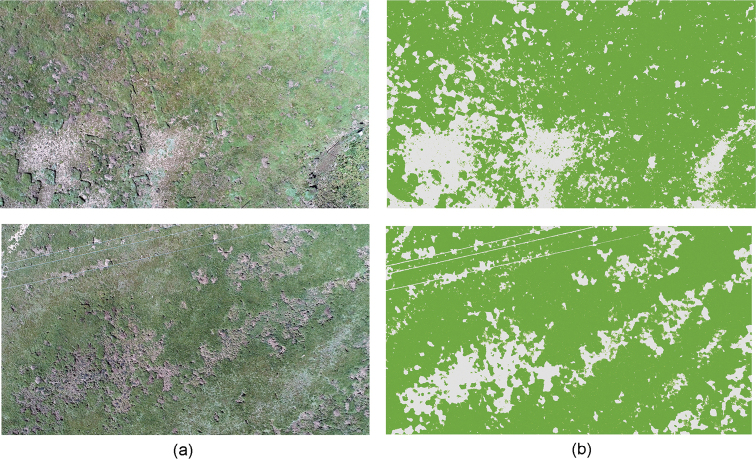
The area of barren land in the study area **a** original remote sensing images of the study area, and **b** images processed to identify the area of barren land.

To verify this conclusion, ArcGIS software (2018) was used to classify barren land and grassland and calculate the area of barren land in the study area. The results are shown in Fig. 7, with both the original picture and the processed image by ArcGIS software. The percentage area of barren land was 18.2%~30.1%, with an average of 23.3%. According to these percentages and the classification criteria proposed by Ma (2006), the grassland in the study area has reached a moderate level of degradation.

## Discussion and conclusion

### Distribution of plateau pikas

The breeding peak of plateau pikas occurs in May and June, with no breeding occurring in middle and late August. In July and August, because of the slow reproduction rate of pikas, interspecies competition, increased precipitation and other factors, the mortality of pikas increased, and the population decreased. The field observations in this study were completed in mid-August, and the average population density of pikas in the study area was estimated to be 144/ha. During the breeding peak of pikas, the average density of pikas in the area could have been much higher than 144/ha.

Plateau pikas were more densely distributed in the environments near the water source and in the gentle terrain. This pattern may have been due to the following reasons: (1) greater vegetation growth near the water source, (2) a shorter distance to the source of drinking water, and (3) the reduced energy consumption and risk of predation associated with drinking water. However, the highest density did not appear at the riverbank, which was the site nearest the water source. This finding may have been due to river flooding in the summer rainy season, which increases the risk of submergence of rodent burrows; in addition, water consumption by predators occurs frequently at this time. The highest population density of pikas occurred at the site 100 m from the riverbank, followed by that 300 m from the riverbank. The growth of vegetation on sunny, steep slopes is poor, and such slopes are not conducive to pika escape from natural enemies. These observations might explain why the density of pikas on the sunny gentle slope was significantly higher than that on the sunny steep slope.

### Behavioral characteristics of plateau pikas

In the areas with higher population densities of pikas (100 m and 300 m from the riverbank), the plateau pikas had low vigilance times, whereas in the area with low densities (the sunny gentle and sunny steep slopes), they alerted each other more frequently. This difference may have been due to the higher individual safety in the areas with higher population density. The numbers of traveling and vigilance activities were highest at the riverbank, where predators frequently drink water; thus, pikas increased their vigilance and traveling behaviors to avoid the risk of predation.

The plateau pika is a diurnal animal, and its foraging behavior exhibited two peaks: 8:00~9:00 and 17:00~18:00. Plateau pikas forage frequently in the early morning and at dusk to reduce energy consumption and the risk of predation. In Dari County, there is abundant sunshine. When the ground temperature is high, the activity of pikas is reduced to reduce energy consumption.
