# ﻿Corrigendum: The distribution and behavioral characteristics of plateau pikas (*Ochotonacurzoniae*). ZooKeys 1059: 157–171. https://doi.org/10.3897/zookeys.1059.63581

**DOI:** 10.3897/zookeys.1086.77554

**Published:** 2022-02-17

**Authors:** Jun Qiu, Cang Ma, Ying-Hui Jia, Jin-Zhao Wang, Shou-Kai Cao, Fang-Fang Li

**Affiliations:** 1 State Key Laboratory of Plateau Ecology and Agriculture, Qinghai University, Xining, 810016, China; 2 State Key Laboratory of Hydroscience & Engineering, Tsinghua University, Beijing, 100084, China; 3 School of Water Resources and Electric Power, Qinghai University, Xining, 810016, China; 4 College of Water Resources & Civil Engineering, China Agricultural University, Beijing, 100083, China

## Abstract

With the help of Wen-Jing Li, the authors realized there was a mistake of surface temperature in the paper above. The surface temperature in the paper refers to the temperature recorded in images which was detected by the temperature receptors of the field infrared camera, instead of the real surface temperature. As there was no shelter to the field infrared camera in alpine meadow grasslands, the temperature of the camera increased rapidly under the sunshine. Thus the conclusion of the preferable temperature for pikas may be around 31~35°C was not right, neither the temperature in Figure 6. As there was no equipment to measure the surface temperature in the observation, it is hard to restore the original surface temperature. The data in Figure 6 can be used to compare the behavior characteristics of plateau pikas at different relative temperature.

The authors would like to declare that the temperature in the paper refers to the temperature recorded in images which was detected by the temperature receptors of the field infrared camera, instead of the real surface temperature. The historic high air temperature in Dari County was 23.2°C (Wang et al. 2018). The temperature of the camera could increase rapidly under the sunshine, so the temperature in the paper can only be taken as a reference of the relative temperature.

The authors would like to give an extra special thanks to Wen-Jing Li, who provides the professional advice and help.

Thanks to the letter by Li (2021), the authors were made aware of a mistake regarding the surface temperature reported in the paper above. The surface temperature reported in the paper refers to the temperature recorded in images detected by the temperature receptors of the field infrared camera, which did not reflect the actual surface temperature. As there was no shelter to the field infrared camera in alpine meadow grasslands, the temperature of the camera increased rapidly under direct sunshine. Thus, the conclusion that the preferable temperature for pikas may be approximately 31~35°C was incorrect, and the temperature reported in Figure 6 was also therefore incorrect. As there was no equipment to measure the surface temperature in the observation, it is difficult to restore the original surface temperature values. The data in Figure 6 should only be used to compare the behavior characteristics of plateau pikas at different relative temperatures.

The authors would like to declare that the temperature in the paper refers to the temperature recorded in images detected by the field infrared camera’s temperature receptors, and does not reflect the actual surface temperature. The historic high air temperature in Dari County was 23.2°C (Wang et al. 2018), so the temperatures cited in the paper can only be taken as a reference of the temperature of the cameras in the sun.

The authors would like to acknowledge Dr. Wen-Jing Li for pointing out this discrepancy in surface temperatures.
